# Paediatrician perspectives and experiences accessing language interpretation services in Paediatric healthcare settings: a POPCORN network qualitative descriptive study

**DOI:** 10.1093/pch/pxag019

**Published:** 2026-04-28

**Authors:** Haowen (Kari) Guo, Ji Yoon Kim, Palak Desai, Mariane Saroufim, Maryam Parvez, Francine Buchanan, Matthew Carwana, Carrie Costello, Carla Southward, Shazeen Suleman, Gita Wahi, Caroline Quach, Manish Sadarangani, Marianne Rufiange, Patricia S Fontela, Peter J Gill, Samina Ali, Thierry Lacaze-Masmonteil, Ananya Banerjee, Patricia Li

**Affiliations:** Centre for Outcomes Research and Evaluation, Research Institute of the McGill University Health Center, Montreal, QC, Canada; Department of Epidemiology, Biostatistics and Occupational Health, School of Population and Global Health, McGill University, Montreal, QC, Canada; Department of Epidemiology, Biostatistics and Occupational Health, School of Population and Global Health, McGill University, Montreal, QC, Canada; Department of Epidemiology, Biostatistics and Occupational Health, School of Population and Global Health, McGill University, Montreal, QC, Canada; Centre for Outcomes Research and Evaluation, Research Institute of the McGill University Health Center, Montreal, QC, Canada; Department of Epidemiology, Biostatistics and Occupational Health, School of Population and Global Health, McGill University, Montreal, QC, Canada; Centre for Outcomes Research and Evaluation, Research Institute of the McGill University Health Center, Montreal, QC, Canada; Department of Epidemiology, Biostatistics and Occupational Health, School of Population and Global Health, McGill University, Montreal, QC, Canada; Office of Patient, Family, and Community Engagement, The Hospital for Sick Children (SickKids), Toronto, ON, Canada; Institute for Health Policy Management and Evaluation, University of Toronto, Toronto, ON, Canada; BC Children's Hospital Research Institute, Vancouver, BC, Canada; Department of Pediatrics, University of British Columbia, Vancouver, BC, Canada; The Children's Hospital Research Institute of Manitoba, Winnipeg, MB, Canada; Bloorview Research Institute, Holland Bloorview Rehabilitation Hospital, Toronto, ON, Canada; Stanford Maternal & Child Health Research Institute, Stanford University, Stanford, CA, USA; Division of General Pediatrics, McMaster Children's Hospital, Hamilton, ON, Canada; Department of Pediatrics, McMaster University, Hamilton, ON, Canada; Faculty of Medicine, University of Montreal, Montreal, QC, Canada; Research Institute, CHU Sainte-Justine, Montreal, QC, Canada; Department of Pediatrics, University of British Columbia, Vancouver, BC, Canada; Vaccine Evaluation Center, BC Children's Hospital Research Institute, Vancouver, BC, Canada; Research Institute, CHU Sainte-Justine, Montreal, QC, Canada; Department of Epidemiology, Biostatistics and Occupational Health, School of Population and Global Health, McGill University, Montreal, QC, Canada; Department of Pediatrics, Faculty of Medicine, McGill University, Montreal, QC, Canada; Office of Patient, Family, and Community Engagement, The Hospital for Sick Children (SickKids), Toronto, ON, Canada; Institute for Health Policy Management and Evaluation, University of Toronto, Toronto, ON, Canada; Department of Pediatrics, University of Toronto, Toronto, ON, Canada; Department of Pediatrics, Faculty of Medicine & Dentistry, University of Alberta, Edmonton, AB, Canada; Women and Children's Health Research Institute, Faculty of Medicine & Dentistry, University of Alberta, Edmonton, AB, Canada; Department of Pediatrics, Cumming School of Medicine, University of Calgary, Calgary, AB, Canada; Department of Epidemiology, Biostatistics and Occupational Health, School of Population and Global Health, McGill University, Montreal, QC, Canada; Centre for Outcomes Research and Evaluation, Research Institute of the McGill University Health Center, Montreal, QC, Canada; Department of Epidemiology, Biostatistics and Occupational Health, School of Population and Global Health, McGill University, Montreal, QC, Canada; Department of Pediatrics, Faculty of Medicine, McGill University, Montreal, QC, Canada

**Keywords:** Language interpretation services, Communication barriers, Health inequity, Barriers in paediatric healthcare, Interpreters, Newcomers

## Abstract

**Objectives:**

Language inaccessibility can perpetuate inequities in health outcomes, service utilization, and patient and caregiver satisfaction in paediatric care. The use of professionally trained interpreters through language interpretation services (LIS) is associated with improved safety and health outcomes, but barriers to accessing LIS persist. Paediatricians' perspectives and experiences are critical to understand how LIS is accessed and could be improved to enhance paediatric care for linguistically diverse populations. Accordingly, we sought to explore paediatricians' experiences accessing LIS.

**Methods:**

A qualitative descriptive study was conducted among paediatricians working within children's hospitals across Canada. Semi-structured interviews were conducted from July 2023 to January 2024, continuing until data saturation was reached. We analyzed the interview data employing thematic analysis guided by the Levesque Framework for healthcare access.

**Results:**

We interviewed 20 paediatricians from 12 of 16 Canadian children's hospitals. All hospitals had some form of LIS (in person or remote), with increased access to remote interpreters since the COVID-19 pandemic. All participants identified the existence, availability, and importance of LIS. They understood the necessity of LIS to facilitate clear communication as part of providing holistic and equitable healthcare. Barriers, including lack of timely services, paediatrician compensation, training, and standardization, persist. Paediatricians suggested measures such as enhanced training and ongoing investments to improve access to interpreters, thereby enhancing equitable healthcare and promoting inclusion.

**Conclusion:**

Canadian paediatricians identified continued barriers, and the need for increased training and clear policies to standardize language need identification and LIS use. Continuous investment and quality improvement are also needed.

## Background

Canada is a country comprised of rich and diverse linguistic communities. According to the 2021 census, 8.5 million (23.2%) people in Canada speak a language other than the two official languages (English or French) at home (1).

Language is an important social determinant of health (2,3). Children and families who are unable to communicate in their preferred language other than English or French (PLOEF) face substantial health inequities with respect to their quality of care, health outcomes, service utilization, and satisfaction (4–6). Previous research has shown increased use of nonindicated intervention, higher risk of emergency department (ED) returns, and poorer experience with the access/coordination of care for families with PLOEF (7–9). Linguistic marginalization is also interconnected with social isolation, racism, and lack of social support within paediatric healthcare (10). For paediatricians communication barriers lead to increased stress, lower provider satisfaction, and less confidence in the provision of high-quality and safe care (3,11–13).

To address communication barriers, language interpretation may be provided by family members or friends, healthcare personnel, community volunteers, and professional medical interpreters (14). In the paediatric setting, the child patient may also be compelled to act as the interpreter for their parents, although the Canadian Paediatric Society strongly recommends against this (3,15). Use of untrained interpreters in general poses a risk to confidentiality, clarity and accuracy, making the use of formal language interpretation services (LIS) provided by medically trained professional interpreters the preferred option (4,16). However, despite the availability of LIS, it remains substantially underused, with a previous reported utilization rate of below 50% and even lower during busy periods or in lower-acuity ED cases (17).

Previous studies have reported patients' or caregivers' experiences in accessing LIS (6,16,18). As paediatricians are also users and gatekeepers of LIS, understanding their experiences provides another point of view on facilitators and barriers to the LIS currently in use. Additionally, paediatricians have a responsibility to take strategic steps to improve hospital outcomes for patients and families with PLOEF (19). Therefore, we conducted a qualitative descriptive study to explore paediatricians' perspectives and experiences in accessing LIS in children's hospitals, to identify ways to improve access and better support their use of LIS.

## Methods

This qualitative descriptive study was part of Paediatric Outcome imProvement through COordination of Research Networks (POPCORN), a research platform involving all children's hospitals in Canada (20). This study received approval from McGill University Faculty of Medicine and Health Sciences Research Ethics Board (REB# 23-05-050).

### Participants and sampling

We recruited paediatricians (general and sub-specialists) who worked in one of 16 Canadian children's hospitals affiliated with the POPCORN network, spoke English or French, and provided consent to participate. Participants were recruited via email through purposive sampling, followed by snowball sampling, to capture a variety of paediatricians working in different hospital settings (inpatient wards, ED, intensive care unit, and outpatient). Interviews were conducted until inductive thematic saturation (i.e., no new codes or themes emerged from additional interviews, and themes were sufficiently developed). Each participant was offered a $25 gift card.

### Positionality statement

The research team comprised graduate students, paediatricians, researchers, and parent partners with expertise in public health, paediatrics, qualitative methodology, knowledge mobilization, and patient engagement. Many team members identify as 1st or 2nd generation migrants or within racial and ethnic minority groups, and speak languages in addition to English and/or French. The majority work closely with children and families in the hospital setting. These professional and lived experiences informed the study throughout, including the interpretation of data.

### Data collection

The first author (HG), trained in qualitative research, conducted in-depth semi-structured interviews in English on Zoom from July 2023 to January 2024. Each interview lasted an average of 60 minutes. The interview guide covered multiple topics, including the perceived importance of LIS, availability of LIS, identification of language needs, experiences accessing LIS, and recommended improvements for LIS. Interviews were audio recorded, transcribed, and transcriptions were manually verified by the first author for accuracy. Field notes, with observations, experiences, and reflections of the first author, were documented after the interview.

### Data analysis

**Figure 1:**
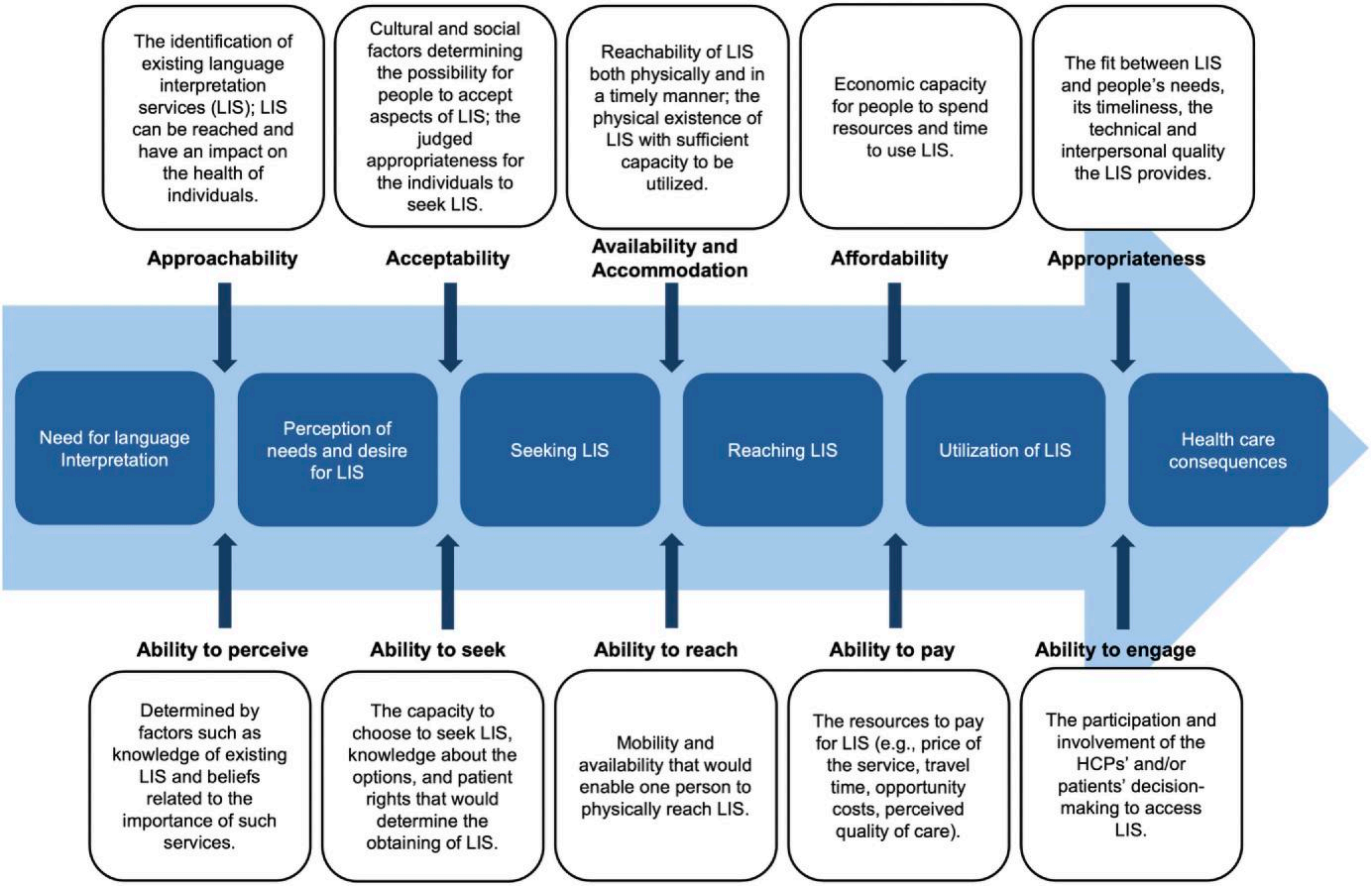
Dimensions of the Levesque framework (22) adapted for language interpretation services. The dimensions above the central arrow represent dimensions from a systematic/supply perspective; the dimensions below the central arrow represent dimensions from an individual/demand perspective. Boxes above or below each dimension represent the descriptions of each dimension.

Data collection and analysis occurred concurrently. Data were analyzed through thematic analysis as described by Braun and Clarke (21) using NVivo 12 software (QSR International Pty Ltd. Version 12). All transcripts were read and coded inductively by the first author to develop the initial codebook. All coded transcripts were reviewed and verified by a second researcher (MS, PD, and MP) to support consistency in coding. Any discrepancies in coding were resolved through a discussion with a third researcher (JK, PD, or MP). The codebook was refined as the interviews and analyses proceeded. Data were coded and expanded into themes to describe paediatricians' experiences accessing LIS. Themes were reviewed (inspected for overlap and merged where applicable), and mapped onto the dimensions of the Levesque framework (Figure 1) (22). Team meetings were held to reach a consensus on the interpretation of data, the development of emerging and final themes, and their alignment with the Levesque framework.

We selected the Levesque framework because it offers a comprehensive, empirically grounded conceptualization of healthcare access, developed from a synthesis of key access-to-care frameworks. It provides a multi-level perspective that integrates healthcare system dimensions (e.g., approachability, availability, affordability) with individuals' abilities to perceive, seek, reach, and engage with care along the healthcare trajectory (Figure 1). In the current study, the “individual” was the paediatrician.

Results of the study were distributed for member-checking among participants to ensure an accurate representation of perspectives and enhance the credibility of study findings.

## Results

Twenty paediatricians from 12 (out of 16) children's hospitals in the POPCORN network, across seven provinces in Canada, participated in the study (Table 1). Participants (90%) worked in critical care (20%), emergency medicine (25%), and as paediatric hospitalists (45%). Most participants were female (60%), identified as white (60%), and were between 40 and 49 years of age (55%).

**Table 1:** Demographic data of a total of 20 participants recruited.

Demographic variable	n (%)
Sex Assigned at Birth	
Female	12 (60%)
Male	8 (40%)
Age Group	
20 to 29 years	≤5
30 to 39 years	≤5
40 to 49 years	11 (55%)
50 to 59 years	≤5
60+ years	≤5
Paediatric Departments	
General Paediatrics	9 (45%)
Paediatric Critical Care	≤5
Paediatric Emergency	≤5
Other Paediatric Specialties	≤5
Outpatient clinics	11 (55%)
Race/Ethnicity	
Asian	≤5
Mixed Race/Ethnicity	≤5
South Asian	≤5
White	12 (60%)
Provinces	
Alberta	≤5
British Columbia	≤5
Manitoba	≤5
Nova Scotia	≤5
Ontario	7 (35%)
Quebec	≤5
Saskatchewan	≤5
Number of Languages Spoken by Paediatrician	
1	12 (60%)
2	≤5
>2	≤5

Six major themes were constructed from the coded transcripts applying the Levesque framework, with each theme explored with subthemes (Table 2).

**Table 2:** Themes, subthemes, and exemplar quotes identified through the thematic analysis of interviews.

Theme/Subtheme	Quote
**Theme 1: Approachability and Ability to Perceive**	
** Identification**	1. “[…] there's not a standardized question down there [emergency department] of how they asked whether someone needs an interpreter […] So out of emerge, it's kind of luck of the draw that someone asked, someone tried to find out. And then if so, did they put in the chart.”—Interview 3
** Knowledge**	2. “[…] we would bring the family and the interpreter into the clinic room and do our interviews with the family using the interpreter in person. And then during COVID, it basically moved to sort of a more telephone-based interpreter service […]. And then just in the last few months, they've introduced this new app called [app name].”—Interview 11
** Importance**	3. “I think it's important because we, as providers have a, not only […]. a general ethical imperative to provide care in a language that a family can understand. But […] a moral one […] sort of duty of our profession to be able to provide that care, to prestart provide interpreter assistance when required.”—Interview 5
**Theme 2: Acceptability and Ability to Seek**	
** Clear Communication**	4. “If they (the parents) have any concerns or any questions they were not able to clarify with us, and I could see now also if they are not able to clarify those concerns around the health of the kid, they are more stressed out, and more confused. […] but when we use the [name of the interpreter service] and explain them, then they feel much better because then they understand it.—Interview 9
** Equitable Healthcare**	5. “I think it's tied very deeply to an understanding of equity and diversity. So when we recognize that we are obligated to provide the exact same care to people, regardless of what language they speak, when they walk through the door, but it's not their fault, and they should not be disadvantaged or punished in any way, for speaking a different language or being socioeconomically disadvantaged gender, […] That's when we have actually provided proper care, because right now, it is inequitable care.”—Interview 10
** Cultural Safety**	6. “And often the interpreters that we work with, most often they act a little bit as cultural brokers, [the interpreter] saying [to the patient] like, ‘the doctor said that, but I don't know you understand perfectly’ or, like [the interpreter saying to the] doctor, ‘you said that, they answered this, I don't think they got it, or maybe they understood this, or I feel like there's maybe some discomfort here […].’ So that's super helpful.”—Interview 16
	7. “[…] we definitely rely on the interpreters to use language that is culturally appropriate. […] But I also think that we probably understand that not all the interpreters can do that. For example, […] (patient family and the interpreter) (a)re both speaking [a language], but it doesn't mean that their cultural background's the same. And so there, it doesn't mean that the interpreter can necessarily interpret the cultural nuances or can necessarily support us in interpreting those. […] I think it's important for us to not necessarily rely on them.”—Interview 18
**Theme 3: Availability and Accommodation, Ability to Reach**	
	8. “I would say (barriers include) availability (of certain languages). For instance, I have a family who's from [country] and speaks two languages, both of which are not available through our local [in-person LIS], and not available in [virtual LIS].”—Interview 2
	9. “[…] within the past, maybe a year and a half or so, we actually have video translators, which I kind of love because you actually get some human interaction through a video. And it's also readily available. The downsides of it are sometimes there is nobody available. So then you end up having to switch to just audio. And they only have one of those per pod. And there's 12 patients' rooms per pod. So or often it's [device] in use, you can't find it, because someone's left it in a room where they use it a lot. And so again, it becomes an availability thing.”—Interview 12
	10. “The other thing that I think is, so the only time families can access interpretation for healthcare is when the healthcare provider requested. So if they wanted to call us, they can't access an interpreter to do that; we need to access an interpreter to reach out to them. So it really limits family's ability to kind of initiate any care […] And even in the hospital, if families want an interpreter and it hasn't been offered, they really have no kind of direct recourse to access it themselves.”—Interview 8
**Theme 4: Affordability and Ability to Pay**	
** Funding/Budget**	11. “I think what makes everything happen, money. So money obviously showed up from somewhere. And the need had been identified. They were funded; somebody paid for it. And you have to pay by the minute, so it's a running budget. So obviously they developed a budget, and someone in administration recognized that this was worth spending money on.”—Interview 10
** Time and Compensation**	12. “Because it (using LIS) takes a lot of time, and I have a lot to do. There's long waitlists, and there's not enough bodies to provide care. So I think it (the challenges to use LIS) is more things like that.”—Interview 16
	13. “And then from the physician, billing perspective, and this is a very real concern. I know I have voiced it, I'm pretty sure my colleagues have voiced it to the [province] Medical Association. There's no extra billing codes for that time for physicians. And so where you could have billed for two, at least one other, if not two other patients in that time. You're now, you've billed only for this, seeing that one patient, even though you've spent double or triple the amount of time that that billing code is supposed to represent. […]”—Interview 19
	14. “[…] From a practical perspective, […] when I see a code, it says, translate required in the comment, someone's registered at triage and says translator required, I purposely will often not sign up for that patient directly. […] I know automatically, that if I'm going into that room, it's probably going to be at least half an hour. […] so I often will not go in and directly see patient until one my learners to sign up, which is okay. But yeah, […] that (extra time spent) is a bit of a barrier. And then, even for learners, it's gonna slow down how many patients they get to see, and when they get to the next patient and all those things. And we don't staff extra for this. We still are the same staff. So […] the more of the patients that require the translation services, if it's unexpected, everything else runs at the speed of expecting that everyone speaks English. And so, all of a sudden, you get two or three of these patients that taken double or triple the time and, it has effects in terms of how fast you see them, the next patient, department flow, all those things, wait times. So there's domino effects on this.”—Interview 19
	15. “But my anecdotal experience is that the residents and students who work with me know that this is my interest. And so, the joke is that, if I'm on call with them, they'll be like, you better use an interpreter, and you better document which language and so on. […] I find more of our residents are interested in this population and doing a lot of the advocacy themselves, which is great.”—Interview 2
	16. “[…] when there's the physical device in the room, it prompts people who walk in the room, even for a short encounter, […] to then use translation (LIS) because it's physically staring at them; […] Where's the phone, […] it was a bit more of a process. […] there's been a number of my colleagues who don't have anything typically to do with new Canadian families' advocacy (advocacy of using LIS for new Canadian families) who say, “Oh, [the new virtual LIS] is great, I never used to use a translator before”. So we're really trying to just encourage the use of it to demonstrate the ongoing need, because we've always known there's a need, and we think that […] translation services in general have been underused.”—Interview 2
**Theme 5: Appropriateness and Ability to Engage**	
** Confidentiality**	17. “Even though we trust, and we tell our interpreter to tell them, and we tell them that the interview is confidential, that what's in the office, stays in the office, but there are some I have come across some families who are worried that maybe they might know the interpreter or the interpreter might know people they know because they attend the same community area or the same mosque or the same center of some sort. And they might be worried that information will be divulged.”—Interview 1
	18. “I'm always worried when it's a friend that they're going to hear sensitive information. Extreme examples, if you're talking about a sexually transmitted infection, or something like that, and now your friend who's not signed a privacy agreement or anything else, confidentiality agreement, knows that you have a sexist transmitted infection, or there's domestic abuse involved or something like that, and can spread it to the community.”—Interview 17
** Quality**	19. “I certainly have had experiences where I know that the translation is terrible, or not up to par.”—Interview 2
** Situational**	20. “[…] the personal preference for me depends on the acuity of the patient. If something is acute, then we go ahead with whatever is available that probably we go ahead with the iPad […], because I know it will be really fast and there will be a person even though he won't be in [the city], […] but they will be able to provide that immediate access to the language interpretation. If there are elective family meetings or updates, then I prefer to have an interpreter come in person in the unit. So that we can sit around and then you know, discuss around and because it is more comforting for the family to see a person who speaks their own language, and you know and provide the timely feedback as a person.”—Interview 9
	21. “I think that's when they're using it the most, because again, you can imagine a nurse with one patient who's critically unwell, that nurse or respiratory therapist is literally in that room, at least, for a 12-hour shift, I would say at least 50 to 60 times doing something, it's just not physically possible to call into the translator every single time. So Google Translate has been super helpful to do that”—Interview 13
	22. “Although occasionally, I don't mind if there is a trusted family relative who I think is actively and appropriately translating. Part of the reason for that, as well as I feel like if they have somebody like that, who's hearing all my discharge instructions, who's hearing everything, it's almost a second check for that family is to someone's gonna go home with them that also knows what I said and can reiterate things. Whereas with the [name of LIS], it's great while you're there, but that person is not going home with them as a resource to what that doctor says about this.”—Interview 19
**Theme 6: Priority and Ability to Improve**	
** Training**	23. “There needs to be training. Everyone needs to know how to use them (interpreter services), and how to utilize them, and best utilize them. So not just oh, every time you see family, you pick up the phone, you call [the interpreter service]. That's, sure, but how do you do it? Where do you do it?”—Interview 3
	24. “I think that the training around that (presuming it's okay not to use LIS if the family is trying to speak English), and the awareness around that needs to change, such that it's assumed the family should have access to an interpreter, unless they tell you, they really don't want it. But it should be something that we attempt to do to optimize healthcare for everybody.”—Interview 18
** Standardization of Care**	25. “It's fairly simple. I mean, (the ideal LIS)'s free, accessible, no friction, no delays, access to any language that the family feels comfortable using, translate to a language I feel comfortable using from medical, in medical terms. So anytime for anyone free, without friction, without having to call anyone (to get access to LIS), without having to ask for permission, without fear of retribution for this.”—Interview 20
	26. “I think things that we can do, we can standardize identification, like standardize at school, what your language preference is, and making it clearly seen. So it's just part of the history, it's just part of your medical record.”—Interview 12
** Investment**	27. “I always wished that there would be a system where there would be sort of an interpreter device in every room, and anybody could decide that interpreter was needed, including the family and just access one kind of in real time. So I think it feels a bit what we're doing right now with interpretation feels a little bit, not tokenistic, but just not a priority. And there's been not enough investment in it.”—Interview 8

### Theme 1: approachability and ability to perceive

Participants recognized communication barriers, were aware of existing LIS, and understood its potential to bridge language gaps. This theme highlights participants' ability to identify when LIS should be implemented during clinical encounters.

#### Identification

Participants named multiple points throughout the healthcare trajectory where identification for language needs can occur: at the time of registration, upon initial interaction with patients, and during patient transfers. However, the practice is not consistently “standardized” and the need for interpreters can sometimes be unknown to participants before their first encounter with patients (**Quote 1,** Table 2).

#### Knowledge

Participants were aware of at least one format of LIS offered (in person, via phone, or video) at their paediatric centre. The COVID-19 pandemic shifted access from in-person interpreters to interpretation via technology with audio or video (**Quote 2**). Informal interpretation was also possible and included family, friends, and online translator programmes (e.g., Google Translate).

#### Importance

Participants believed that LIS were important because they played key roles in obtaining accurate information, obtaining consent, and maintaining patient safety. Some participants also voiced ethical and moral imperatives to provide language-concordant care for patients with language needs (**Quote 3**).

### Theme 2: acceptability and ability to seek

Participants collectively supported the use of LIS to address communication barriers. Their acceptance was shaped by cultural and social factors, such as promoting equity and ensuring cultural safety of patients.

#### Clear communication

Professional medical interpreters facilitated bi-directional communication between paediatricians and their patients. Participants understood that caregivers may be in distress and may seek to communicate their concerns, needs, and questions “clearly” (**Quote 4**). In turn, participants wanted to ensure clarity in their own communications with patients and caregivers regarding the health condition(s) and care plan.

#### Equitable healthcare

Many participants sought LIS as it promoted equitable and inclusive healthcare to diverse patients, which they valued and regarded as a means to reduce systemic racism in paediatric healthcare (**Quote 5**).

#### Cultural safety

Many participants also chose to seek LIS to support cultural safety in the provision of care with respect to patients' language preferences and rights. They described interpreters often serving as “cultural brokers” to communicate information in culturally relevant ways that address the needs of the patient (**Quote 6**). However, some participants also expressed that it's important not to rely on interpreters for cultural brokering, as interpreters may not feel comfortable or be trained for this, and shared languages don’t always equate with shared cultures (**Quote 7**).

### Theme 3: availability and accommodation, ability to reach

As mentioned above, at least one form of LIS was available at all participating children's hospitals. However, paediatricians' ability to access the service in a timely manner and the ability for LIS to accommodate the varying needs of paediatricians and patients was based on factors such as the availability of in-person or remote interpreters and languages (e.g., less commonly encountered languages), and of the devices (e.g., tablet or phone) (**Quotes 8 and 9).**

Further, participants reported that patients and caregivers do not have the ability to reach or request for LIS, and that the provider must initiate access to an interpreter (**Quote 10**).

### Theme 4: affordability and ability to pay

The affordability of LIS for the hospital was not posed as a challenge. However, restrained resources such as time and compensation were barriers to the use of LIS.

#### Funding/budget

Most participants reported that there was a budget available to implement LIS, which reflected support for such services from the administration or leadership at some level (e.g., hospital, provincial, federal) (**Quote 11**). However, most of them were not aware of the funding details.

#### Time and compensation

Additional time was usually required to access and use LIS. Paediatricians were often under time pressure to move things along due to long waitlists or short staff in the hospital (**Quote 12**). Moreover, in some provinces, under the fee-for-service remuneration models, there was no compensation for paediatricians who spent extra time communicating with patients and families using LIS. Some participants mentioned that the constant time pressure and the lack of additional compensation for the extra time spent can be barriers to care for patients and families with language needs (**Quotes 12** and **13**).

Although the additional time required to work with LIS families may be a barrier for some paediatricians, for example, in high acuity settings where they may avoid these families (**Quote 14**), other paediatricians with a strong interest in LIS populations may be biased toward actively advocating for them and modeling LIS use for trainees (**Quote 15**). Easily accessible LIS, such as in-room virtual interpreter devices within quick reach, were preferred because they save time and may encourage greater use by paediatricians when caring for families with language needs (**Quote 16**).

### Theme 5: appropriateness and ability to engage

Appropriateness highlights the fit between language services and the needs of the patient and paediatrician. This theme encompasses when, which, how, and why services are provided, and, importantly, the paediatricians' ability to engage in effective communication.

#### Confidentiality

Participants reported that patients' privacy and confidentiality must be preserved and conserved during the use of LIS. Most of them expressed a preference to use trained interpreters who are obliged to maintain confidentiality compared to family members as interpreters. However, their patients have raised concerns about confidentiality breaches from trained interpreters as they may be members of their communities, given their shared language (**Quote 17**).

In parallel, participants worried about using family members as informal interpreters, as it also posed a risk to confidentiality since they were not required to sign a privacy agreement (**Quote 18**).

#### Quality

A few participants reported that the interpretation lacked quality (e.g., mistranslation) despite being from a formal LIS (**Quote 19**).

#### Situational

Participants expressed that certain formats of LIS were more appropriate for certain settings. For example, acute situations require readily accessible formats, such as those provided online/over the phone, whereas in-person LIS may be preferred when this could be arranged for non-urgent visits or multi-disciplinary meetings. They felt that the presence of an in-person interpreter could provide additional emotional support to the family (**Quote 20**).

Participants also provided examples of where informal interpretation was fitting for the needs of the paediatricians and caregivers, whether it is Google Translate or a family member during different points of care (**Quotes 21** and **22**).

### Theme 6: priority and ability to improve

Paediatricians offered suggestions at the organizational level on how to prioritize and improve access to LIS to enhance equitable healthcare. This additional theme falls outside of the Levesque framework.

#### Training

Training for paediatricians on the importance, acceptance, and effective utilization of LIS to support patients from diverse linguistic backgrounds was strongly recommended (**Quotes 23** and **24**).

#### Standardization of care

Participants reported that LIS should be standardized and that they should always have access to an interpreter at any time of the day when language presents as a barrier to communication (**Quote 25**).

Further, participants advocated that the preferred language of communication of all patients should always be asked during intake and charted to ensure timely access to LIS when required (**Quote 26**).

#### Investment

Due to the barriers and limitations identified, participants advocated for continued investment and resource allocation for LIS and as an integral part of the hospital’s overall paediatric services (**Quote 27**).

## Discussion

This qualitative study provides insight into the experiences of paediatricians in accessing and using LIS across Canadian paediatric hospitals. Based on the Levesque framework, our results illustrated that paediatricians were aware of the existence, availability, and importance of LIS. They sought LIS to enable clear communication with families, equitable healthcare, and cultural safety. Despite the importance of LIS and its continuous improvement over the past years, our findings suggest that many barriers persist. Participants offered recommendations to reduce barriers and improve LIS to enhance health equity.

A key barrier to LIS usage highlighted by our participants was the lack of standard identification of language preferences among patients and families. Our work supports previous studies reporting the lack of standardized processes for identifying language preferences, providing interpretation, and documenting language data, which have led to inconsistent recognition and documentation of language needs (23,24). Clinics with standardized language identification have reported better communication and higher provider satisfaction (25). Screening and documentation of language preferences could be achieved with basic screening questions as piloted by Murphy *et al.* (19) in the primary care setting and through electronic health record (EHR) solutions, as suggested by our participants and the literature (23–25).

Research has shown that implementing structured fields in the EHR for patients preferred languages and interpretation needs pose minimum burden. Regulations or EHR systems that mandate filling out preferred languages and flag interpreter needs may further facilitate the standard identification of language preferences (26). An EHR-based record for language and interpreter needs may also track language frequency, LIS expenses, and billings for paediatricians (27). However, to implement standardized language identification and promote LIS use, clear policies and guidelines are essential. A policy in Melbourne led to a 317% increase in interpreter requests, decreased readmission rates, and decreased length of stay (28). Implementing standardized practices in Canada can mitigate disparities in LIS provision driven by provider attitudes or perceptions, and support equitable and culturally safe care (29).

Paediatricians highlighted the limited access to LIS due to shortage of interpreters, technology, and languages available for interpretation (e.g., less prevalent languages). Inadequate LIS can lead to delayed and suboptimal care, decreased treatment adherence and use of preventive services, increased hospital and ED visits, and lead to poor health outcomes (4). However, the use of communication technologies, such as 24-hour on-demand professional audio and video interpretation, can improve LIS availability in healthcare settings (30). Further, monitoring their use through quality improvement metrics would be needed to ensure effectiveness and guide ongoing optimization.

Time was another major barrier to accessing LIS reported by our participants. Consistent with this finding, a narrative scoping review of 21 studies identified time as the most frequently reported factor influencing paediatricians' use of LIS (31). Jones et al. similarly highlighted time constraints as a key barrier, particularly in situations requiring urgent care or when workload did not allow sufficient time to schedule an interpreter (29). These time demands have contributed to negative attitudes toward patients and families with language needs, further restricting their LIS use (12). However, in our study, paediatricians with a particular interest in LIS demonstrated greater willingness to spend the additional time needed to access LIS. Their active use of LIS also appeared to contribute to growing interest among residents, highlighting the importance of role modelling and demonstrating the value of LIS in clinical practice.

Lastly, mandatory training of healthcare providers in using LIS would enhance access across the dimensions of Levesque's framework. Our participants and prior research emphasized the importance of such training, which is largely lacking in Canada (32). Training of paediatricians can increase awareness of the availability and importance of LIS. This can support *approachability* and help paediatricians better *perceive* the importance of LIS. Furthermore, training can foster a culture of *acceptability* and encourage providers to *seek* LIS among diverse populations with differential language needs. *Availability and accommodation* through efficient and timely use of in-person or remote interpreters can also be ensured through training. Finally, it can strengthen *appropriateness* by promoting accurate, patient-centred communication, essential in paediatric care. Overall, training paediatricians when and how to use LIS can enhance their confidence and efficiency in using LIS, further ensuring equitable care for patients and families (33,34).

### Limitations

Our study is one of the first to report on the perspectives and experiences of providers accessing LIS in Canadian paediatric healthcare. While the findings contribute to our understanding, they may not be generalizable. First, data were only collected from paediatricians' perspectives and therefore do not reflect the experiences of other healthcare providers (e.g., nurses), caregivers, or interpreters, who may also provide valuable insight into the process and efficacy of LIS. Most of our participants identified as white, and all of our participants worked in a tertiary care children's hospital, in the inpatient or outpatient settings. Therefore, these findings may not be generalizable to community paediatric settings, which may have little to no LIS resources and funding. We also did not have the perspectives of paediatricians who had never or rarely accessed LIS.

## Conclusion

Paediatricians identified numerous barriers to accessing LIS and gaps in provider training. Despite these challenges, they recognized the critical role of LIS in delivering equitable and culturally safe paediatric care. Strengthening policies to standardize language identification and LIS use, alongside enhanced training and compensation for paediatricians, can improve the timely use of interpretation, ultimately supporting better care for all patients and families with diverse language needs.
